# Harbor porpoise distribution and habitat use in the Northern California Current over three decades

**DOI:** 10.7717/peerj.21021

**Published:** 2026-04-09

**Authors:** Dawn R. Barlow, Craig S. Strong, Barbara Muhling, Leigh G. Torres

**Affiliations:** 1 Marine Mammal Institute, Department of Fisheries, Wildlife, and Conservation Sciences, Oregon State University, Newport, Oregon, United States; 2 Crescent Coastal Research, Crescent City, California, United States; 3 Fisheries Collaborative Program, Institute of Marine Sciences, University of California, Santa Cruz, Santa Cruz, California, United States; 4 Southwest Fisheries Science Center, National Oceanic and Atmospheric Administration, La Jolla, California, United States

**Keywords:** Habitat, Harbor porpoise, Northern California current, Upwelling, Distribution, Density surface modeling

## Abstract

In the dynamic marine environment, mobile predators adapt their distribution and habitat use in alignment with regional biogeographic features that confer predictable prey resources. However, long-term changes in environmental conditions may challenge the resilience and persistence of regionally adapted populations. In this study, we investigated how densities of a marine predator, the harbor porpoise, vary relative to habitat characteristics and whether they respond to large-scale fluctuations in environmental conditions. Vessel-based visual surveys were conducted between 1992 and 2022 in the nearshore waters of the Northern California Current. We developed stock-specific density surface models to examine harbor porpoise distribution relative to environmental covariates and predict density over time. This enabled us to identify long-term, stable spatial hotspots, and evaluate inter-annual fluctuations in density relative to ocean basin-scale climate indices and annual upwelling phenology. Harbor porpoises exhibited stock-specific habitat preferences reflecting regional biogeography. Predicted densities were higher in shallow, nearshore waters, with closer proximity to capes and estuaries, and in response to intermittent upwelling. Harbor porpoise density within our nearshore study region was significantly higher during years with longer and stronger upwelling seasons, and lower during El Niño conditions. Taken together, these findings emphasize the importance of regionally guided population assessment and management, and careful consideration of the potential impacts of climate change and anthropogenic pressures on this sensitive nearshore species and the habitats they rely on in the Northern California Current.

## Introduction

In the ocean, resources are patchy, and spatiotemporal alignment between mobile predators and critical habitat that supports foraging is vital to the persistence of predator populations (Hyrenbach, Forney & Dayton, 2000; Benoit-Bird, 2024). As environmental conditions fluctuate due to global climate change and prey resources shift in response, mobile predators must adapt to changing habitat and prey availability. Populations that are resident to particular areas are often adapted to local biogeography (Shuai et al., 2018; Barlow et al., 2023). As a result, regional adaptations may influence the resilience of predator populations to changing environmental conditions and shifting prey. Therefore, understanding whether and how marine predators display regional biogeographic adaptations in their distribution, habitat preference, and density, is key for both advancing fundamental ecological knowledge and informing regionally guided population assessment and management.

The Northern California Current (NCC) is a productive eastern boundary current system that supports a rich diversity of species (Bograd et al., 2009). In the spring and summer, upwelling-favorable winds from the north generate a net movement of surface water offshore resulting in an influx of cold, nutrient-rich subsurface water into the photic zone. The nearshore waters of the NCC (within ~5 km of shore) are further shaped by complex bathymetric features including sloping soft bottom substrate, rocky reefs, and kelp forests (Romsos et al., 2007), creating a rich mosaic of habitat for numerous invertebrate, fish, seabird, and marine mammal species (Oregon Department of Fish and Wildlife, 2016). Large estuarine tidal flows and the Columbia River plume provide additional nutrient inputs (Hickey & Banas, 2008; Bidlack et al., 2021), and prominent cape features and offshore banks create areas of recirculation that retain nutrients and support recruitment at the base of the food web (Barth, Pierce & Castelao, 2005; Castelao & Barth, 2005; Morgan, Fisher & Largier, 2011). At a regional scale, the timing, intensity, and duration of coastal upwelling can vary substantially between years and between biogeographic areas (Bograd et al., 2009), and these variations in upwelling phenology have implications for coastal predators (Oestreich et al., 2022; Pirotta et al., 2024). At an even larger scale, the NCC is also influenced by ocean basin-scale climate patterns such as the Pacific Decadal Oscillation (PDO) and the El Niño Southern Oscillation (ENSO), which can dictate overarching warm or cool conditions across the region, with ecosystem-wide impacts across trophic levels (Mantua et al., 1997; King et al., 2011; von Biela et al., 2016; Peterson et al., 2017; Shimabukuro et al., 2023). The interplay between how nearshore marine predators select habitat based on fine-scale static and dynamic features and the influence of large-scale environmental patterns on population abundance over decadal time scales warrants further investigation, with the potential to elucidate multi-scale drivers of species occurrence and persistence in this dynamic ecosystem.

One predator inhabiting the NCC is the harbor porpoise (*Phocoena phocoena*), a small, cryptic odontocete occupying waters <200 m depth (Barlow et al., 1988; Carretta, Taylor & Chivers, 2001). They typically exhibit site fidelity (Forney et al., 2017) and have minimal migratory or genetic exchange between biogeographic regions of the NCC (Chivers et al., 2002, 2007; Morin et al., 2021). Therefore, harbor porpoises are likely adapted to local habitat characteristics throughout their range. Harbor porpoises require high foraging rates to meet basic energy demands (Wisniewska et al., 2016) due to their small body size and cool temperate habitat, making frequent and predicable access to prey imperative for survival. Thus, their habitat preferences likely reflect regional drivers of reliable prey resources. In the NCC, harbor porpoise distribution has been associated with areas of nearshore upwelling and elevated productivity (Tynan et al., 2005). Stomach content analyses of stranded or bycaught harbor porpoises along the west coast of North America recorded numerous consumed species, frequently including Pacific herring (*Clupea pallasi*), northern anchovy (*Engraulis mordax*), Pacific sardine (*Sardinops sagax*), market squid (*Doryteuthis opalescens*), smelt (*Osmeridea*), and hake (*Merluccius productus*), among others (Gearin et al., 1994; Byrd, 2001; Toperoff, 2002; Nichol et al., 2013). However, precise dietary preferences across their range remain largely unknown due to sampling challenges. Defining the preferred habitat characteristics of harbor porpoises in the NCC may facilitate improved understanding of how static and dynamic features yield enhanced foraging opportunities for harbor porpoises throughout their range.

**Figure 1:**
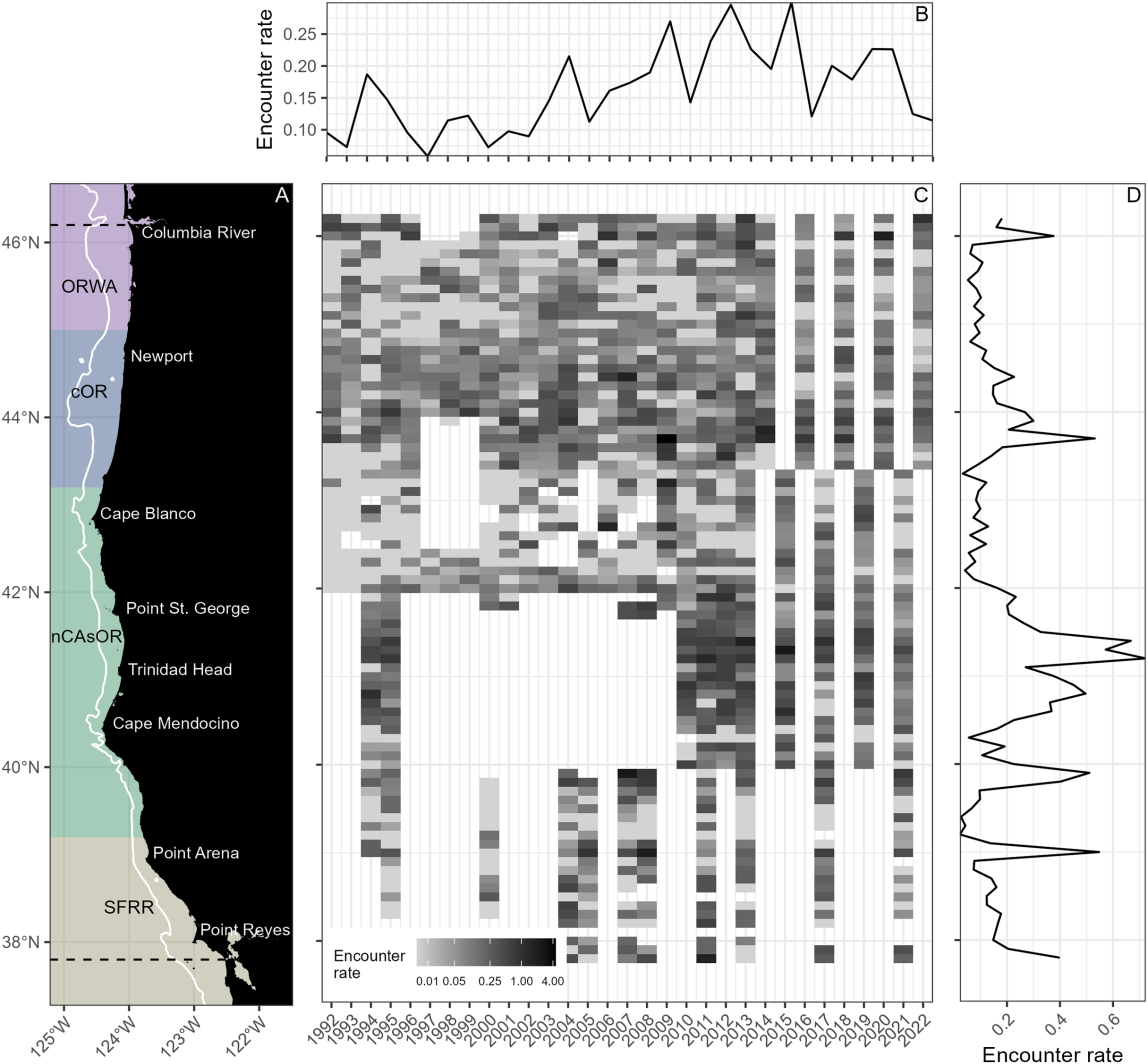
Harbor porpoise encounter rate. (A) Map of the study area, with the north and south boundaries of the study area denoted by the dashed lines. Colored panels show the latitudinal boundaries for each harbor porpoise stock, and the white line shows the 200 m isobath, considered the offshore boundary for each stock. Major placenames denoted. (B) Mean harbor porpoise encounter rate (porpoises/kilometer surveyed) summarized by year, across all latitudes. (C) Harbor porpoise encounter rate summarized by year (x-axis) and 1° latitude bin (y-axis), with darker cells indicating higher encounter rate (note: color ramp visualized on a log scale, sigma = 0.01). Any cell that is filled (colors ranging between gray and black) represents a latitude bin and year for which there was survey effort; empty white cells represent lack of survey effort in that year and latitude bin. (D) Mean harbor porpoise encounter rate summarized by 1° latitude bin, across all years.

Four harbor porpoise stocks are currently recognized in the NCC (Figs. 1, S1) based on genetics and distribution patterns (Morin et al., 2021; Forney, Benson & Becker, 2023; Carretta et al., 2024): Northern Oregon-Washington Coast (ORWA; 2022 abundance = 22,074, CV = 0.39), Central Oregon (cOR; 2022 abundance = 7,492, CV = 0.42), Northern California-Southern Oregon (nCAsOR; 2022 abundance = 15,303, CV = 0.57), and San Francisco-Russian River (SFRR; 2017 abundance = 7,777, CV = 0.62). None of these four stocks are listed as threatened or endangered under the Endangered Species Act nor as depleted or strategic under the Marine Mammal Protection Act. Harbor porpoise numbers off California have rebounded after the prohibition of coastal set-gillnet fishing, which was a large source of bycatch (Forney et al., 2021). However, they are still threatened by fishery-related mortalities, anthropogenic noise, pollution, and reduced prey availability (Forney et al., 2017; Carretta et al., 2024). As an acoustically sensitive species, harbor porpoises are susceptible to auditory injuries at low noise levels (Lucke et al., 2009; Brandt et al., 2018), and can be temporarily displaced by construction and pile driving operations (Carstensen, Henriksen & Teilmann, 2006; Tougaard et al., 2009; Brandt et al., 2018), seismic surveys (Thompson et al., 2013; Pirotta et al., 2014), and offshore wind turbines (Koschinski et al., 2003), potentially forcing them into areas with reduced habitat quality or increased threats (Forney et al., 2017). As human use of the marine environment continues to accelerate (Jouffray et al., 2020), comprehensive analysis of harbor porpoise habitat use is warranted. While broad-scale analyses of harbor porpoise distribution patterns have produced abundance estimates, examined population recovery, and informed stock assessments (Forney et al., 2021), there is a paucity of information on ecological drivers of harbor porpoise distribution, including adaptation to regional habitat characteristics. Moreover, as a mobile, nearshore predator, the harbor porpoise may be a valuable model for understanding how regionally adapted predator populations respond to variable environmental conditions, and how habitat preferences may render each stock vulnerable in the face of natural and anthropogenic threats.

In this study, we compile 31 years of standardized, nearshore surveys in the NCC (Fig. 1) between 1992 and 2022 to model harbor porpoise habitat use and density in the nearshore portion of their range, providing insights into stock-specific harbor porpoise adaptations to fine-scale habitat characteristics. The long-term perspective also enables us to examine fluctuations in harbor porpoise density over time relative to annual upwelling phenology and large-scale climate oscillations.

## Materials and Methods

**Figure 2:**
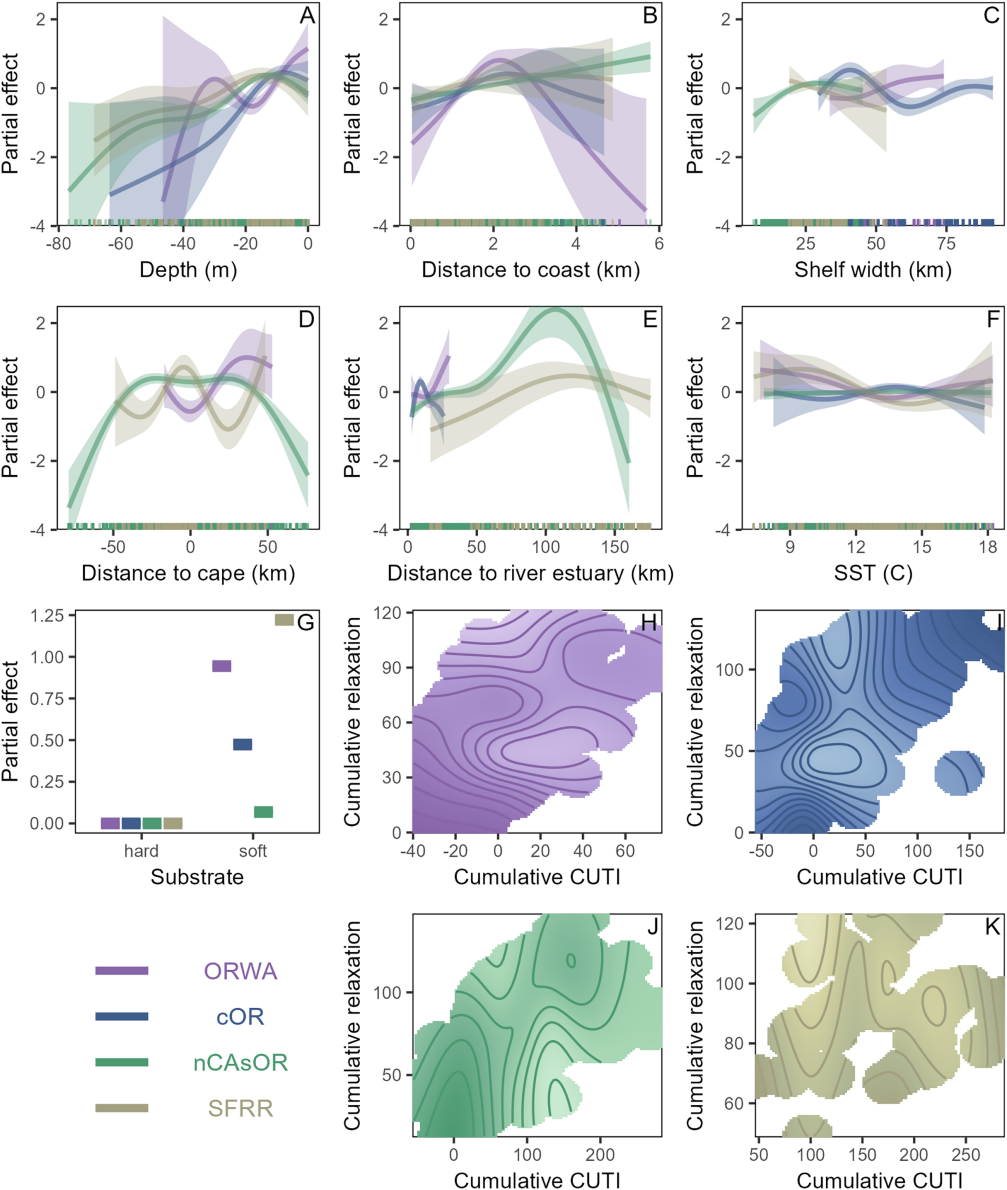
Functional relationships between harbor porpoise density and habitat covariates. Partial response plots illustrating the functional relationships identified by the harbor porpoise density surface models, with colors designating the relationships for each nearshore (<5 km) stock region (legend in bottom left corner). (A–F) Functional response curves for smoothed predictor variables. (G) Functional response for categorical predictor variable. (H–K) Functional response for the interactive effect of cumulative CUTI and cumulative relaxation for each stock region, with light coloration representing a strong positive partial effect and dark coloration representing a strong negative partial effect; areas with no color represent environmental conditions that did not occur in the dataset. Note the different axis ranges for (H–K).

Our study area spans nearshore waters (<5 km from shore) between the Columbia River and San Francisco Bay (37.8°N–46.2°N, Fig. 2). Field research was designed to monitor at-sea abundance of Marbled Murrelets (*Brachyrampus marmoratus*) (Raphael et al., 2007; Mciver et al., 2021). The coastline was partitioned into 20 km-long primary sampling units (PSUs). Each PSU included four 5 km-long “inshore” transect segments, running parallel to the coast at four randomly selected distances <1,500 m from shore north of Coos Bay. South of Coos Bay, the 5 km-long “inshore” segments extended out to 2 km from shore. Each PSU also included an “offshore” transect, conducted on a diagonal transect from the inshore boundary out to 5 km (north of Coos Bay) or 3 km (south of Coos Bay), with a randomized starting point for each PSU sample (Fig. S2). The multiple, randomized distances from shore of the “inshore” transects and the diagonal orientation of the “offshore” transect ensures survey coverage across nearshore environmental gradients. Trained observers recorded all seabirds and marine mammals; we focus on harbor porpoise observations. While surveys covered only the inshore portion of the known harbor porpoise range, which extends offshore to depths up to 200 m, prior research has documented a skewed distribution toward shallow, nearshore waters <50 m (Forney, Benson & Becker, 2023). Nonetheless, we recognize that our study area—limited by the Marbled Murrelet survey design—and any interpretation of observed patterns refer only to harbor porpoise density in nearshore waters, and do not capture offshore distribution, or movement between inshore and offshore areas.

Vessel-based surveys were conducted between annually between May–August from 1992–2022, aboard a 21-foot “Boston Whaler” model vessel following line-transect, distance sampling methods (Buckland et al., 2015). Two observers scanned between the bow and the beam on port and starboard, respectively, while the driver maintained a speed of 10 kt. Observation conditions, including Beaufort sea state (BSS) and an ordinal “sightability” metric summarizing overall conditions, were recorded continuously. Observers were trained in visual distance estimation prior to each season, with weekly calibration testing. At all harbor porpoise sightings, group size and perpendicular distance to the trackline were recorded. Encounter rate (number of harbor porpoises per km effort) was summarized by year across the study area (37.8°N–46.2°N), by 0.1° latitude bin across all years, and by year and 0.1° latitude bin.

### Density surface modeling

#### Detection function

Distance sampling methods use distances between the sampler (vessel) and observation (harbor porpoise group) to estimate probability of detection at various distances from the trackline, using a detection function constructed from the distance data (Buckland et al., 2015). We fit candidate detection functions using the ‘Distance’ R package (Miller et al., 2019), which included no covariates, BSS, sightability, or both as covariates, each tested with both half-normal and hazard-rate keys, and compared using Akaike’s information criterion (AIC). The detection function was used to estimate effective strip width (ESW) under different covariate conditions. In subsequent model fitting, ESW was applied to all survey segments given the conditions at the time.

Due to sampling design, we could not empirically estimate availability bias of harbor porpoises at the surface. We therefore were required to assume porpoises are unlikely to be missed and do not exhibit vessel avoidance behavior, though we acknowledge this is a substantial assumption of harbor porpoise behavior. Additionally, we assume unaccounted-for availability bias is constant and will therefore not affect overall trends, *i.e*., abundance may be underestimated by an unknown but (assumed) constant amount.

#### Environmental data

We modeled harbor porpoise abundance relative to static and dynamic environmental variables with the potential to influence harbor porpoise foraging opportunities, thereby driving their distribution and habitat use patterns (Table 1, Fig. S3). Static layers were computed at a 1 km resolution. Depth was obtained from the general bathymetric chart of the oceans (GEBCO, 15 arc-second resolution), and high-resolution coastline information was obtained from OpenStreetMap. These two datasets were used to generate continuous layers representing depth, distance from shore, shelf width, and distance from prominent capes. Shelf width and distance from capes were included because of their role in retaining upwelled nutrients, enhancing nearshore productivity, and influencing prey aggregation (Castelao & Barth, 2005; Morgan, Fisher & Largier, 2011). Shelf width was measured as the shortest distance from the coast to the 200 m isobath. To identify prominent capes, a 200 km continuous smooth was applied to the high-resolution coastline. Capes were designated as locations where the distance between the smoothed and high-resolution coastline exceeded 5 km (Barlow, Strong & Torres, 2024). Subsequently, a continuous layer representing distance to nearest cape was created, where negative values indicated locations north and positive values represented locations south of capes (Fig. S3). As proximity to river estuaries may enhance harbor porpoise foraging (Holdman et al., 2019), location and size of river estuaries were extracted from the Pacific Marine and Estuarine Fish Habitat Partnership Estuary Points dataset (PSMFC GIS, 2018), limited to river estuaries >300 hectares (encompassing most rivers but excluding small creeks), and a continuous layer representing distance to nearest estuary was generated. Benthic substrate was classified as hard or soft based on multibeam sonar mapping conducted, processed, and provided by the Active Tectonics and Seafloor Mapping Lab at Oregon State University (Goldfinger et al., 2014), and the Seafloor Mapping Lab at California State University Monterey Bay (SFML, 2014).

**Table 1:** Harbor porpoise habitat covariates.

Metric	Description	Source
Depth	Bathymetric depth (m)	GEBCO bathymetry
Distance to coast	Distance to coastline (km)	Open street map high resolution coastline
Shelf width	Distance from the coast to the 200 m isobath (km)	GEBCO bathymetry, Open street map high resolution coastline; single distance value per latitude applied across longitudes
Distance to cape	Distance and direction to the nearest prominent cape (km)	Distance to the nearest prominent cape feature; positive values indicate location is south of headland. Cape locations defined as coasline features that differ >5 km from a 200 m smoothed coastline (high resolution coastline from Open Street Map)
Distance to estuary	Distance to nearest riverine estuary >300 hectares (km)	Pacific Marine and Estuarine Fish Habitat Partnership
Substrate	Hard or soft benthic substrate	Active Tectonics and Seafloor Mapping Lab, Oregon State University (Oregon), and Seafloor Mapping Lab, California State University Monterey Bay (California)
SST	Sea surface temperature (°C)	Optimal Interpolation SST (0.25 degree resolution), downloaded from ERDDAP
Cumulative CUTI	Daily cumulative upwelling index	Cumulative daily smoothed CUTI, calculated for each 1° latitude bin
Cumulative relaxation	Cumulative number of days where the daily upwelling index value falls below the “relaxation event” threshold	Relaxation events are defined as days when the CUTI value falls below the mean CUTI during the upwelling season (spring transition through end of upwelling, calculated following Bograd et al. (2009)). The relaxation threshold is computed separately for each latitude bin, and the mean is calculated across the full study period (1992–2022)

**Note:**

Static and dynamic environmental covariates included in the harbor porpoise surface models, along with the source of the data and relevant information on how the metrics were computed from the data.

Three dynamic variables were included: sea surface temperature (SST), upwelling, and relaxation (intermittent cessation of upwelling). Daily SST was sourced from the optimal interpolation SST (OISST, 0.25° resolution) product, which integrates observations from various platforms (satellites, ships, buoys, Argo floats). Although spatially coarse, this cloud-free product covers the full temporal range of the surveys, and is highly correlated with finer-scale Multi-spectral Ultra-high Resolution SST within our study area during the 20-year period when the products overlap (Fig. S4). Coastal upwelling and relaxation were derived from the daily Coastal Upwelling Transport Index (CUTI, 1° latitude bins). CUTI estimates vertical flux using regional sea surface height, wind stress, and mixed layer depth *via* regional ocean reanalysis (Jacox et al., 2018). To mitigate the influence of extreme spikes, a 10-day running average smoothing filter was applied to daily CUTI (Oestreich et al., 2022). Subsequently, a daily cumulative upwelling index was computed for each 1° latitude bin. While the spatial scale of CUTI is coarser than the survey data, it is the finest scale upwelling index available, and captures substantial temporal variability (Jacox et al., 2018). Cumulative CUTI was used to define the upwelling season, identified as the period between the spring transition index and the end of the upwelling season, following Bograd et al. (2009). Cumulative CUTI was recalculated starting at the spring transition. Mean CUTI within the upwelling season was then computed across the 31-year study period for each latitude bin. Relaxation is an ecologically important phenomenon that occurs when upwelling-favorable winds subside, leading to retained nutrients, enhanced productivity, and prey aggregation (Menge & Menge, 2013; Benoit-Bird, Waluk & Ryan, 2019; Dawn et al., 2025). Recent evidence shows the importance of alternation between upwelling and relaxation for the distribution and morphology of nearshore predators in the NCC (Barlow, Strong & Torres, 2024; Pirotta et al., 2024). We characterized “relaxation events” as days when CUTI fell below mean CUTI during the upwelling season for that latitude bin (Barlow, Strong & Torres, 2024; Dawn et al., 2025). Cumulative relaxation was calculated for each day as the cumulative number of days classified as relaxation events since the spring transition.

#### Spatial model fitting

Harbor porpoise observations were summed, and observation conditions were averaged by survey segment. Environmental data were extracted at the segment centroid location for all static layers and SST; cumulative CUTI and cumulative relaxation were assigned to segments by date and latitude bin. Segment length was predetermined by the Marbled Murrelet survey design; while most were 5 km, variation in segment length was accounted for in model fitting.

We fit spatial models by harbor porpoise stock to account for local adaptation to regional habitat characteristics (Morin et al., 2021). Our study area spanned the southern portion of the ORWA stock range, the full latitudinal range of the cOR and nCAsOR stocks, and the northern majority of the SFRR stock range (Fig. S1). Stock boundaries extend to the 200 m isobath (Carretta et al., 2024; Fig. 1), whereas surveys were conducted within 5 km of shore. We thus refer to harbor porpoise stocks but acknowledge that the stock area extends offshore of waters surveyed in our study.

Density surface models (DSMs) are a method for deriving spatially explicit estimates of abundance by modeling species occurrence patterns while accounting for detection probability (Miller et al., 2013). After fitting the detection function to the distance sampling data, a spatial model is constructed from the segmented observation data incorporating the ESW of each segment as an offset, accounting for covariates included in ESW estimation. The spatial component of the DSMs was fit using generalized additive models (GAM), semi-parametric regression models capable of accommodating non-linear relationships through smoothing functions (Wood, 2017). GAMs were fit for each stock with a quasi-Poisson distribution and log link function. The response variable was number of harbor porpoises per segment, and predictor variables included smoothed terms for depth, distance to coast, shelf width, distance to cape, distance to river estuary, and SST. For cOR where prominent capes are scarce, distance to cape was omitted to prevent spurious ecological inferences. Substrate was incorporated as a parametric term. Additionally, a smoothed interaction term between cumulative CUTI and cumulative relaxation was included (2D thin plate regression spline). To reduce overfitting, smoothed terms were restricted by setting the number of knots to k = 5, while the interaction term was set to k = 15. Variable selection employed a shrinkage approach, adding an extra penalty to each smoother and penalizing non-significant variables to zero (Marra & Wood, 2011). DSMs were implemented in the ‘dsm’ R package (Miller et al., 2022).

### Assessment of long-term patterns in harbor porpoise abundance

We used the DSMs to predict daily harbor porpoise abundance across a 5 km grid between the coastline and 5 km from shore. In addition to abundance, density was also computed for each grid cell by dividing predicted abundance by the grid cell area. Daily predictions were produced between 15 May–30 August from 1992–2022 for each stock. As predictions to non-analogous conditions (*i.e*., environmental conditions never measured during surveys) can lead to unrealistic predictions (Bouchet et al., 2019), days with high extrapolation in the dynamic predictors were excluded using the extrapolation detection (ExDet) tool in the R package ‘dsmextra’ (Bouchet et al., 2020), by removing days containing grid cells with ExDet >1.15 or <−0.15. To examine spatial patterns across the whole study period, mean harbor porpoise abundance and density were calculated for each grid cell across all predictions, with corresponding uncertainty (coefficient of variation; CV), identifying any long-term spatial hotspots in harbor porpoise abundance. To examine temporal patterns across the study area, daily predicted harbor porpoise abundance was summed across all grid cells to produce a daily abundance estimate within the study area for each stock. Then, mean predicted daily abundance, density, and CV were calculated for each year to obtain annual abundance and density estimates by stock. We acknowledge here that the annual estimates are from the period when surveys occurred (May–August), and do not capture intra-annual differences.

### Large-scale environmental correlates of annual harbor porpoise density

Annual harbor porpoise density anomalies were calculated for each stock by subtracting the long-term mean predicted density from the annual mean predicted density for that stock, and then dividing the centered values by the standard deviation. This standardization expresses anomalies in units of standard deviation from the mean, allowing for direct comparison of relative variability among stocks with differing baseline densities. Positive anomaly values represent years with above-average densities within our study area for each stock, and negative values represent below-average densities.

We evaluated the relationships between harbor porpoise density anomaly and several large-scale environmental correlates known to influence regional biological productivity in the NCC. Two ocean basin-scale indices were considered: the PDO and the Multivariate ENSO Index (MEI) were both summarized during the spring (February–May) of each year of the study, thereby characterizing conditions leading up to each survey season (Strong & Duarte, 2023). While the North Pacific Gyre Oscillation (NPGO) was initially considered, its influence is less in the NCC compared to other regions of the California Current and it was therefore not included (Di Lorenzo et al., 2013; Fisher, Peterson & Rykaczewski, 2015). Relationships between annual harbor porpoise density anomaly and regional upwelling phenology were also considered, and several annual metrics were summarized based on the cumulative CUTI, following Bograd et al. (2009). The spring transition index (STI) indicates the day of year of onset of the upwelling season, the annual maximum (MAX) indicates the day of year of maximum upwelling accumulation, the total upwelling magnitude index (TUMI) measures the total cumulative upwelling between the STI and the end of the upwelling season, and the length of the upwelling season index (LUSI) measures the number of days between the STI and the end of the upwelling season. Each of these upwelling phenology indices were computed for each 1° latitude bin of our study region, and then the mean value was computed across the latitudinal range of each harbor porpoise stock. Linear models were fit separately between porpoise density anomaly and each environmental index. Each model included harbor porpoise stock as a factor and allowed for interactions between stock and the environmental index to test whether the relationships varied among stocks. Model parameters were estimated using standard linear regression, and the significance of main and interaction effects was assessed using analysis of variance.

## Results

### Harbor porpoise habitat use

Between 1992–2022, 55,346.2 km of survey effort was completed. Surveys were conducted annually, though effort varied between years (*e.g*., surveys alternating between northern and southern areas after 2014 whereby not all of the study area was covered each year). However, coverage was comprehensive across the study period (Figs. 1, S5). The survey dataset contained 5,594 harbor porpoise observations, totaling 8,156 individuals. Of these observations, 5,396 had distance estimates used to fit the detection function. The truncation distance was set to the 98^th^ percentile of the distances (270.5 m), which removed observations furthest from the trackline that would impact detection function fit while retaining most of the distance data. The selected detection function was fitted with a hazard-rate key and BSS and sightability covariates (Table S1). Estimated ESW ranged between 59–120 m (Fig. S6).

Deviance explained for the DSMs was 20.3% for ORWA, 15.4% for cOR, 15.2% for nCAsOR, and 30.4% for SFRR (Table 2). For all stocks, higher abundance was associated with shallower depths (<30 m), and areas 1–4 km from shore (Figs. 2A, 2B). Shelf width was significant and showed a positive relationship for the ORWA, cOR, and nCAsOR stocks (Fig. 2C). Abundance was higher in areas near (cOR and SFRR) or south of (ORWA) capes (Fig. 2D). The cOR stock showed higher abundances within 20 km of estuaries; others exhibited a positive relationship with distance from estuaries, possibly indicating a geographic effect rather than response to estuaries themselves (Fig. 2E). Porpoises within the nearshore range of the cOR, nCAsOR, and SFRR stocks surveyed were associated with soft bottom substrate (Fig. 2G). The interaction between cumulative upwelling and cumulative relaxation was a significant predictor for all stocks (Table 2), however the relative importance of upwelling *vs* relaxation differed between stocks (Figs. 2H–2K); while the total cumulative upwelling and relaxation differed between stock regions, higher cumulative upwelling seemed to be favored for the ORWA nCAsOR stocks, whereas greater cumulative relaxation seemed to be favored by the cOR and SFRR stocks. SST was significant for all but nCAsOR, with higher abundances associated with cooler temperatures (Fig. 2F).

**Table 2:** Density surface model results. Harbor porpoise density surface model performance metrics and predictor variable contribution for each region. Survey effort is illustrated by the sample size, measured as the number of survey segments for each region. Overall model performance is measured by the deviance explained. Significant predictor variables are denoted by the asterisks (*) associated with their *p*-value in the model, with the number of asterisks denoting the significance level.

	Metric	ORWA	cOR	nCAsOR	SFRR
	Sample size (n segments)	1,610	3,505	3,199	506
	Deviance explained	20.3%	15.4%	15.2%	30.4%
Predictor variable significance (*p*-value)	s (depth)	2.24 × 10^−6***^	7.25 × 10^−5***^	<2 × 10^−16***^	0.002**
	s (distance to coast)	2.15 × 10^−7***^	0.007**	1.92 × 10^−5***^	0.163
	s (shelf width)	0.021*	2.19 × 10^−5***^	5.89 × 10^−4***^	0.150
	s (distance to cape)	0.001**	N/A	<2 × 10^−16***^	1.06 × 10^−6***^
	s (distance to estuary)	0.003**	1.37 × 10^−4***^	<2 × 10^−16***^	0.007**
	Substrate	0.948	7.39 × 10^−5***^	0.007**	2.84 × 10^−5***^
	s (SST)	0.038*	0.065	0.444	0.002**
	s (cumulative CUTI, cumulative relaxation)	0.007**	<2 × 10^−16***^	<2 × 10^−16***^	3.42 × 10^−5***^

**Notes:**

* *p* < 0.05.

** *p* < 0.01.

*** *p* < 0.001.

### Harbor porpoise density

**Figure 3:**
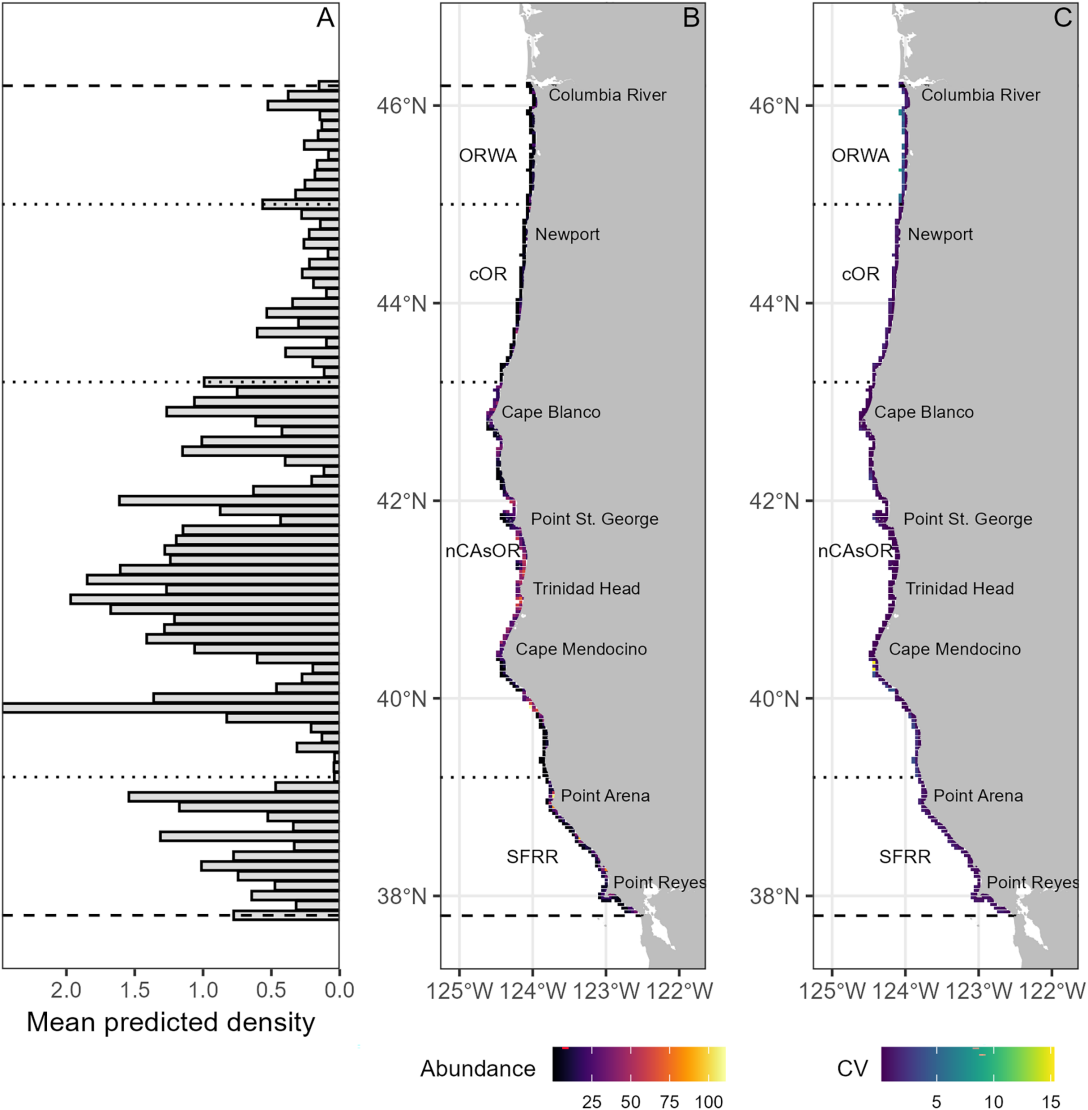
Harbor porpoise spatial distribution. (A) Mean predicted harbor porpoise density (porpoises/km^2^) per 1° latitude bin across the entire study period (1992–2022). (B) Mean predicted daily harbor porpoise abundance per 5 km grid cell, illustrating fine-scale spatial predictions produced by the density surface models for each region. Geographic reference points are denoted in black text. Density surface models were fit and predicted separately for each harbor porpoise stock, denoted by the dashed black dotted lines. (C) Mean associated uncertainty (coefficient of variation, CV) per 5 km grid cell across the entire study period.

Encounter rate varied across years and latitudes (Fig. 1). DSMs predicted harbor porpoise hotspots south of the Columbia River, off Cape Blanco, between Point St. George and Cape Mendocino, off Point Arena, and between Point Reyes and San Francisco Bay (Fig. 3). Overall, spatiotemporal patterns in encounter rate from the surveys and DSM predictions of density aligned, with similar hotspots emerging from both. Low density areas aligned with known stock boundaries (Morin et al., 2021), lending support for these areas as natural population “break points” along a continuous coastline.

Daily predicted harbor porpoise abundance increased slightly between May–August for all but SFRR, which had a higher abundance late June-early July (Fig. S7). Annual predicted abundance within our nearshore study area ranged between 245–673 for ORWA, 408–820 for cOR, 2,730–4,920 for nCAsOR, and 596–1,838 for SFRR, with no consistently increasing or decreasing long-term trends (Fig. S8). Both abundance and density were highest for nCAsOR and lowest for ORWA (Figs. 3, S8). It is important to note that these do not represent stock abundance estimates, as our study area only covers the inshore (<5 km) portion of the range for each stock.

### Large-scale environmental correlates of annual harbor porpoise density

**Figure 4:**
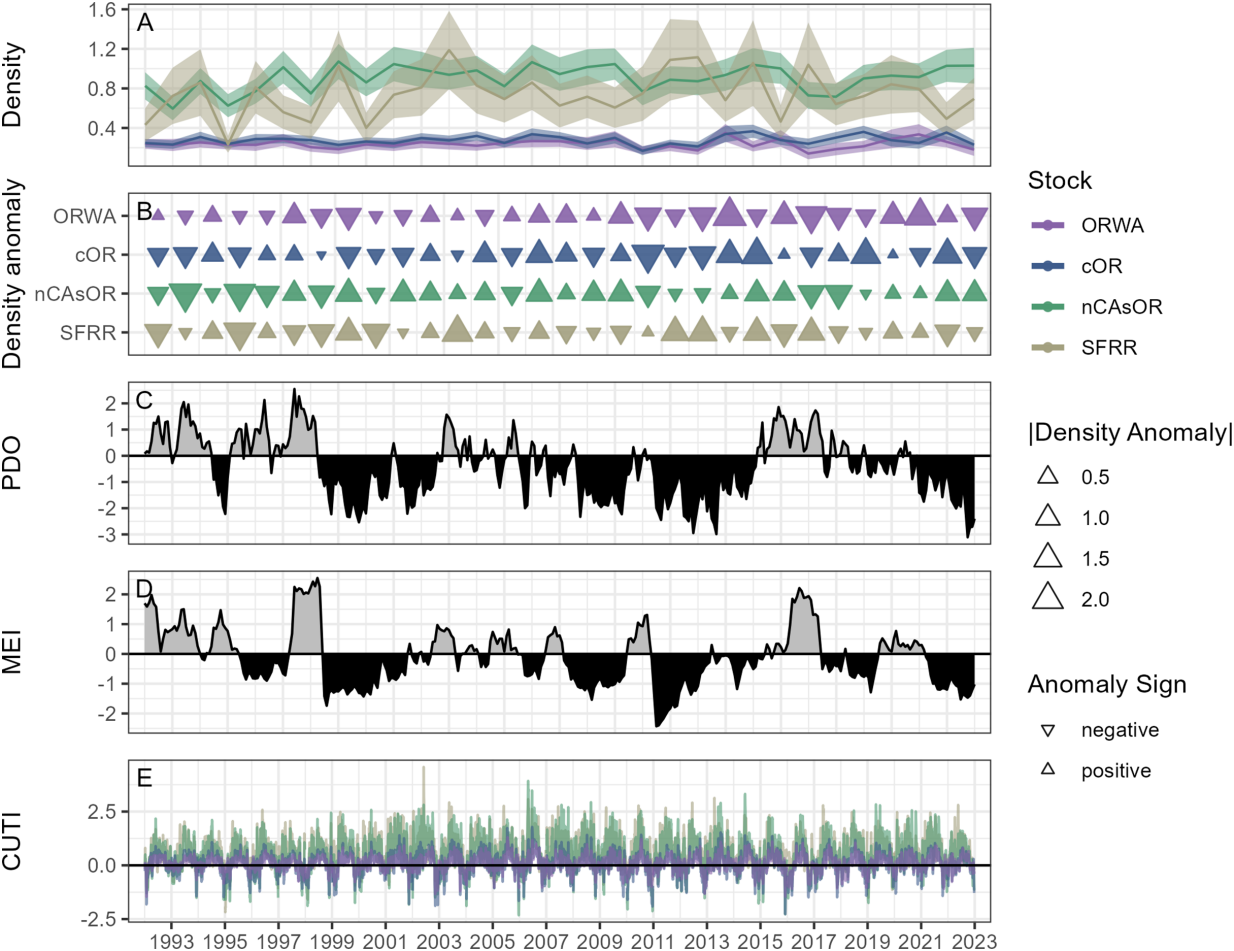
Annual variation in harbor porpoise density and large-scale environmental correlates throughout the study period. (A) Mean annual harbor porpoise density within the study area, with colors denoting the stock and shading showing the standard error resulting from uncertainty in the model predictions. (B) Annual harbor porpoise density anomaly compared to the long-term mean for each year and each stock, with the orientation of the triangle denoting positive or negative anomaly, and the magnitude of the density anomaly shown by the size of each triangle. Triangles are colored by each harbor porpoise stock. (C) Pacific decadal oscillation (PDO), with gray showing positive and black showing negative phases. (D) Multivariate ENSO index (MEI), with gray showing positive and black showing negative phases. (E) Mean coastal upwelling transport index (CUTI). Colors of the lines show the mean CUTI within the latitudinal range of each harbor porpoise stock.

**Figure 5:**
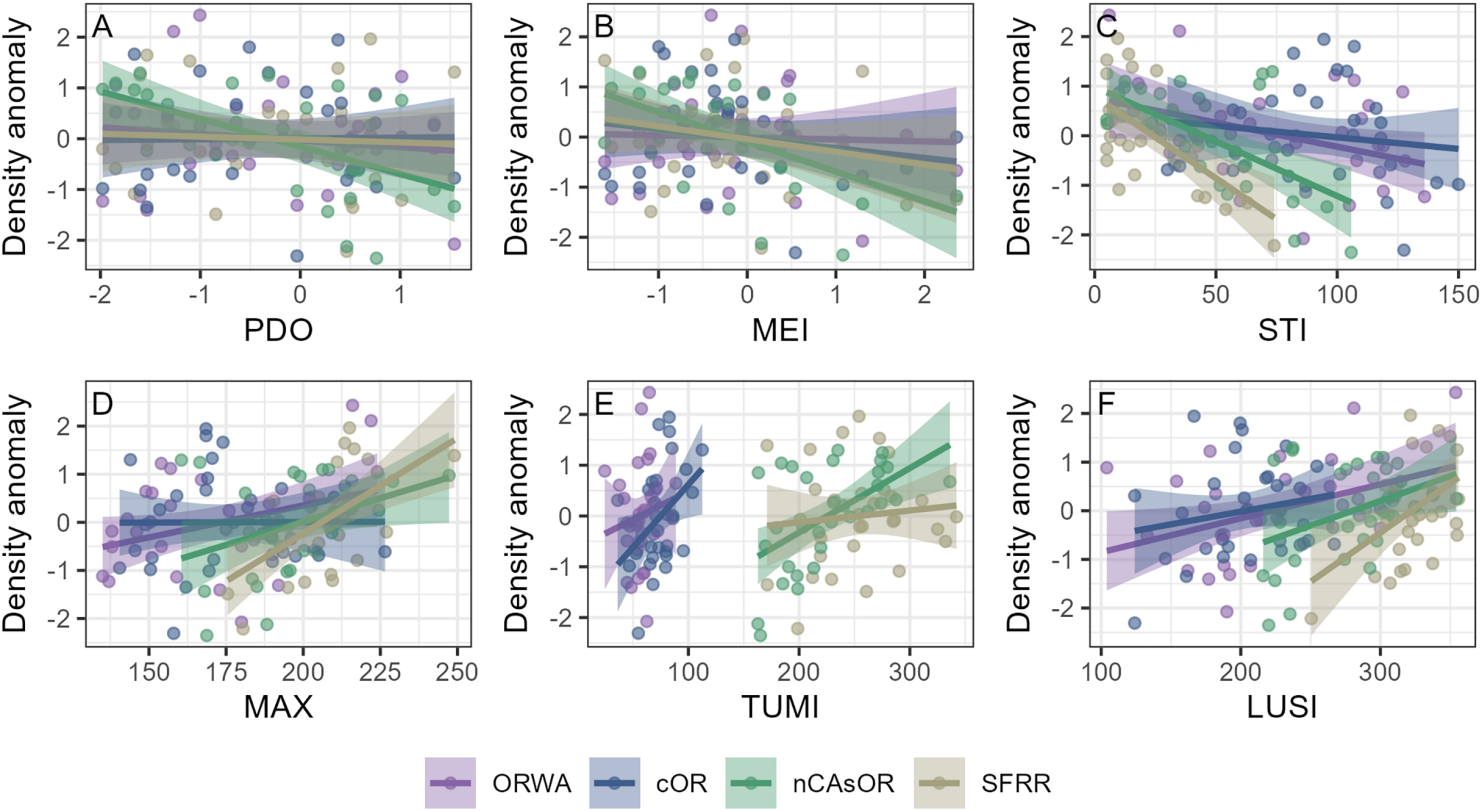
Relationships between annual harbor porpoise density and large-scale environmental correlates. Relationships between harbor porpoise annual density anomaly and (A) Pacific decadal oscillation (PDO), (B) multivariate ENSO index (MEI), (C) spring transition index (STI), (D) day of maximum upwelling accumulation (MAX), (E) total upwelling magnitude index (TUMI), and (F) length of the upwelling season index (LUSI), by harbor porpoise stock.

Linear models revealed relationships between annual harbor porpoise density anomalies and several environmental indices, including variation between stocks (Figs. 4, 5, Table 3). There was no significant relationship between density anomaly and PDO. Density anomaly and MEI showed a weak positive relationship, indicating lower densities during El Niño conditions. In contrast, models examining the upwelling phenology metrics STI, MAX, and LUSI were significant overall, indicating higher porpoise densities under stronger upwelling conditions and a longer upwelling season. All three metrics also showed significant interaction terms, indicating that the strength and direction of these relationships differed among stocks. The interactions between these three upwelling phenology metrics and harbor porpoise stocks showed a north-south pattern (weakest slopes for ORWA and cOR, stronger for nCAsOR, and strongest for SFRR), likely driven by the latitudinal variation in upwelling strength (Fig. 4E). The TUMI model was significant primarily due to among-stock variation rather than a consistent directional effect.

**Table 3:** Relationships between annual harbor porpoise density and environmental indices. Summary of linear model results examining the relationship between harbor porpoise annual density anomaly and environmental indices, including the Pacific decadal oscillation (PDO), multivariate ENSO index (MEI), spring transition index (STI), day of maximum upwelling accumulation (MAX), total upwelling magnitude index (TUMI), and length of the upwelling season index (LUSI). Columns indicate the significance of the overall model and the effects of the environmental index, harbor porpoise stock, and their interaction, based on analysis of variance (ANOVA). Significance levels are indicated as follows: ns = not significant (*p* ≥ 0.1).

	Statistical significance (*p*-value)				
	Overall model	Environmental index	Stock	Interaction	Interpretation
PDO	ns	–	–	–	No significant relationship between PDO and density anomaly.
MEI	0.0997^(.)^	0.0056**	ns	ns	Weak overall model; generally positive relationship across stocks.
STI	4.9 × 10^−5***^	0.00043***	0.011*	0.016*	Strong positive relationships; slope differs among stocks.
MAX	0.0086**	0.0057**	ns	0.055^(.)^	Positive relationships overall; marginally stronger slope in SFRR stock.
TUMI	0.0318*	ns	0.031*	ns	Stock differences in density anomaly; no consistent relationships with TUMI across stocks.
LUSI	0.0148*	0.0139*	0.0345*	ns	Positive relationships with density anomaly; SFRR intercept more negative.

**Notes:**

(.) Marginal (0.05 ≤ *p* < 0.1).

* *p* < 0.05.

** *p* < 0.01.

*** *p* < 0.001.

## Discussion

Using three decades of standardized surveys, we quantitatively described harbor porpoise habitat preferences across the NCC, enabling us to predict their abundance through space and time. Within the nearshore waters surveyed in our study, we demonstrate stock-specific harbor porpoise habitat preferences, indicating regional adaptation to local biogeography. Furthermore, we illustrate how nearshore harbor porpoise density fluctuates with large-scale environmental phenomena that influence regional biological productivity, with densities being consistently higher during years with longer and stronger upwelling seasons, and lower during El Niño conditions. Although our surveys cover waters <5 km from shore (<80 m depth, Fig. 2), harbor porpoises are known to occupy waters <200 m depth (Forney, Benson & Becker, 2023; Carretta et al., 2024). Therefore, our predictions should not be interpreted as stock abundance estimates, but rather as summaries of nearshore spatiotemporal patterns in harbor porpoise density, which elucidate both long-term spatial hotspots and inter-annual fluctuations in density.

While harbor porpoises are known to rely on shallow, coastal waters throughout their range along North America’s west coast (Carretta et al., 2024), we demonstrate additional habitat use patterns throughout the NCC (Fig. 2). While direct observations of foraging were not the focus of this study, the relationships between harbor porpoise density and environmental covariates indicate that habitat selection is likely driven by foraging opportunities. The interactive effect between upwelling and relaxation aligns with how nearshore productivity is maximized when upwelling episodes are interspersed with wind relaxation—termed “intermittent upwelling”—which allows phytoplankton to fully utilize upwelled nutrients (Menge & Menge, 2013). These relaxation events enhance recruitment and aggregation of forage species (Botsford et al., 2006; Barth et al., 2007; Menge & Menge, 2013; Benoit-Bird, Waluk & Ryan, 2019), benefitting nearshore predators (Barlow, Strong & Torres, 2024; Pirotta et al., 2024; Dawn et al., 2025). Along windy, open coastlines, physical features like prominent capes and a wider shelf generate countercurrents that recirculate and retain nutrients and plankton, bolstering the base of the food web (Graham & Largier, 1997; Morgan, Fisher & Largier, 2011), potentially supporting harbor porpoise’ prey. Headland features can also direct tidal flows, enhancing foraging *via* prey aggregation (Johnston, Westgate & Read, 2005; Elliser, MacIver & Green, 2018).

The DSMs revealed how porpoise habitat preferences reflect regional biogeography. The ORWA stock area covered by our study is characterized by the Columbia River plume, reduced upwelling, sandy beaches, and some capes (Fig. S3; Schroeder et al., 2022). ORWA harbor porpoises off Oregon associated with soft substrate, moderate upwelling, and areas south of capes and with wider shelf width that support locally elevated productivity (Barth, Pierce & Castelao, 2005; Castelao & Barth, 2005; Morgan, Fisher & Largier, 2011). While the dynamics of the Columbia River plume are known to influence foraging opportunities for piscivorous predators (Zamon, Phillips & Guy, 2014; Bliss et al., 2024), these effects did not clearly manifest in our analyses, likely because survey effort was focused in nearshore waters south of the main plume. The cOR stock occupies the area where the most river estuaries occur (Fig. S3) that support habitat for forage fish (Thompson et al., 2017), and harbor porpoise abundance was elevated within 15 km N/S of estuaries (Fig. 2). The cOR stock also inhabits waters along a more exposed coastline with fewer capes, and the model revealed a preference for relaxation (Fig. 2I), indicating the importance of wind relaxation to allow nearshore retention in this region. The higher cOR encounter rate and density inshore of Heceta Bank (Fig. 1) aligns with known hotspots for seabirds (Ainley et al., 2005) and baleen whales (Derville et al., 2022), indicating that this bathymetric feature with greater shelf width supports foraging opportunities for multiple predators, including in the nearshore through retention and recirculation (Barth, Pierce & Castelao, 2005; Kudela et al., 2008). The nCAsOR stock occupies a region of strong upwelling along a complex coastline, with recirculation in the lee of prominent capes (Schroeder et al., 2022). In the nCAsOR model, upwelling was more influential than relaxation (Fig. 2J), perhaps because upwelling interacts with these promontories to locally enhance foraging. The SFRR stock occupies a comparatively smaller but variable stretch of coastline, showing preferences for areas near capes, high upwelling and relaxation (Fig. 2K), and lower SST (Fig. 2F). These nuanced stock-specific habitat selection patterns (Fig. 2, Table 2) are supported by genetic evidence for apparent biogeographic boundaries (Morin et al., 2021). Densities differed between stocks indicating that within our study area, nearshore waters of the NCC may be able to support higher porpoise densities in the nCAsOR and SFRR stock ranges. These regional adaptations may influence harbor porpoise response and resilience in the face of environmental change, shifting prey, or anthropogenic impacts.

The relationships between harbor porpoise density anomalies and large-scale environmental phenomena align with what has been shown for other predators in the California Current. El Niño conditions are characterized by an influx of warmer water resulting in a deeper thermocline and reduced primary productivity in the California Current (King et al., 2011). These periods have been shown to impact the reproductive success of several seabirds (Schmidt et al., 2014; Strong & Duarte, 2023) that forage in areas that overlap with harbor porpoise habitat. On a more regional scale, blue whales adjust their foraging behavior and migratory phenology to align with the timing and magnitude of the upwelling season, whereby they will remain on their foraging grounds for longer when a more extended upwelling season supports more feeding opportunities (Oestreich et al., 2022). Although blue whale life history and target prey differ from those of harbor porpoises, their response nonetheless illustrates the role of upwelling phenology to shape the foraging success of another prominent California Current predator. The inter-annual fluctuations in harbor porpoise density we show do not necessarily indicate large swings in stock abundance between years; rather, since our study area is constrained to nearshore waters, our results could reflect porpoises moving to more offshore areas. During El Niño conditions or periods of weaker upwelling, porpoises may expand their search area for suitable prey, leaving a smaller fewer available for detection in our nearshore surveys. Regardless of the mechanism for changing densities, we demonstrate that harbor porpoise density is influenced not only by fine-scale habitat features, but also large-scale environmental phenomena that shape regional biological productivity. With global climate change, these large-scale phenomena are anticipated to shift as well, including more frequent warm water events, changes in vertical and inshore-offshore temperature gradients, and poleward re-distribution of upwelling centers (Rykaczewski et al., 2015; Wang et al., 2015; Santora et al., 2020; Bograd et al., 2023), all of which may contribute to lower nearshore harbor porpoise densities if the patterns in the future.

Harbor porpoises exhibit site fidelity (Elliser, White & Hansen, 2025; Parsons et al., 2025); thus, displacement from critical habitat likely has severe consequences (Forney et al., 2017). While harbor porpoise mortality from fisheries bycatch has been drastically reduced following the ban of coastal set-gillnets (Forney et al., 2021), these sensitive predators are still subject to multiple anthropogenic threats (Carretta et al., 2024). Noise pollution is known to elicit behavioral and distributional responses for harbor porpoises (Tougaard et al., 2009; Pirotta et al., 2014; Forney et al., 2017; Wisniewska et al., 2018; Simonis et al., 2020); therefore, anthropogenic activities such as commercial and recreational vessel traffic, coastal development, and the construction of offshore energy infrastructure pose potential population level impacts if these behavioral responses confer fitness consequences. The NCC supports a number of ports that are hubs for human activity and therefore concentrate impacts on nearshore waters, such as the Columbia River in the north of our study area, Coos Bay in central Oregon, Humboldt Bay in northern California and San Francisco Bay in the south of the study area. So, while threats may be heterogeneously distributed in space and time, all harbor porpoise stocks examined in this study overlap with human activities to varying degrees. Furthermore, given the stock-specific habitat preferences that likely reflect regional adaptation, the ongoing ecosystem-wide shifts in ocean conditions and the forage species community (Thompson et al., 2019; Bograd et al., 2023) have the potential to lead to spatiotemporal mismatch between harbor porpoises and preferred habitat and prey resources.

## Conclusions

In the NCC, harbor porpoises show stock-specific habitat preferences that align with regional biogeography, which can help tailor region-specific management approaches. Additionally, harbor porpoise densities in the nearshore portion of their range increase with longer and stronger upwelling seasons, and decrease under El Niño conditions, likely reflecting the influence of these large-scale patterns on local prey availability. As the expansion of anthropogenic activities puts pressure on coastal habitats (Jouffray et al., 2020) and physical and biological dynamics of productive ecosystems like the NCC shift under climate change (Bograd et al., 2023), there are critical implications for how the adaptation of marine predators to region-specific habitat characteristics may render them vulnerable as conditions change. We therefore emphasize the importance of regional approaches to population assessment and management.

## Supplemental Information

10.7717/peerj.21021/supp-1 Supplemental Information 1 Supplementary figures and tables.

## Additional Information and Declarations

### Data Availability

The following information was supplied regarding data availability:

Raw data and code are available at Figshare:

Barlow, Dawn; Torres, Leigh; Strong, Craig; Muhling, Barbara (2026). Harbor porpoise distribution and habitat use in the Northern California Current over three decades. figshare. Dataset. https://doi.org/10.6084/m9.figshare.27215982.v1.
