# Supplementary material for: Harbor porpoise distribution and habitat use in the Northern California Current over three decades
Source: PeerJ. 2026 Apr 9;14:e21021. doi: 10.7717/peerj.21021 (PMC13070323; doi:10.7717/peerj.21021)
Supplement: Supplemental Information 1 [file peerj-14-21021-s001.pdf]

Harbor porpoise distribution and habitat use in the Northern California Current over three decades

Dawn R. Barlow[1]*, Craig S. Strong[2], Barbara Muhling[3,4], Leigh G. Torres[1]

[1]Geospatial Ecology of Marine Megafauna Lab, Marine Mammal Institute, Department of Fisheries, Wildlife, and Conservation Sciences, Oregon State University, Newport, Oregon, USA

[2]Crescent Coastal Research, Crescent City, California, USA

[3]Fisheries Collaborative Program, Institute of Marine Sciences, University of California – Santa Cruz, California, USA

[4]NOAA Southwest Fisheries Science Center, La Jolla, California, USA

*dawn.barlow@oregonstate.edu

SUPPLEMENTARY MATERIALS

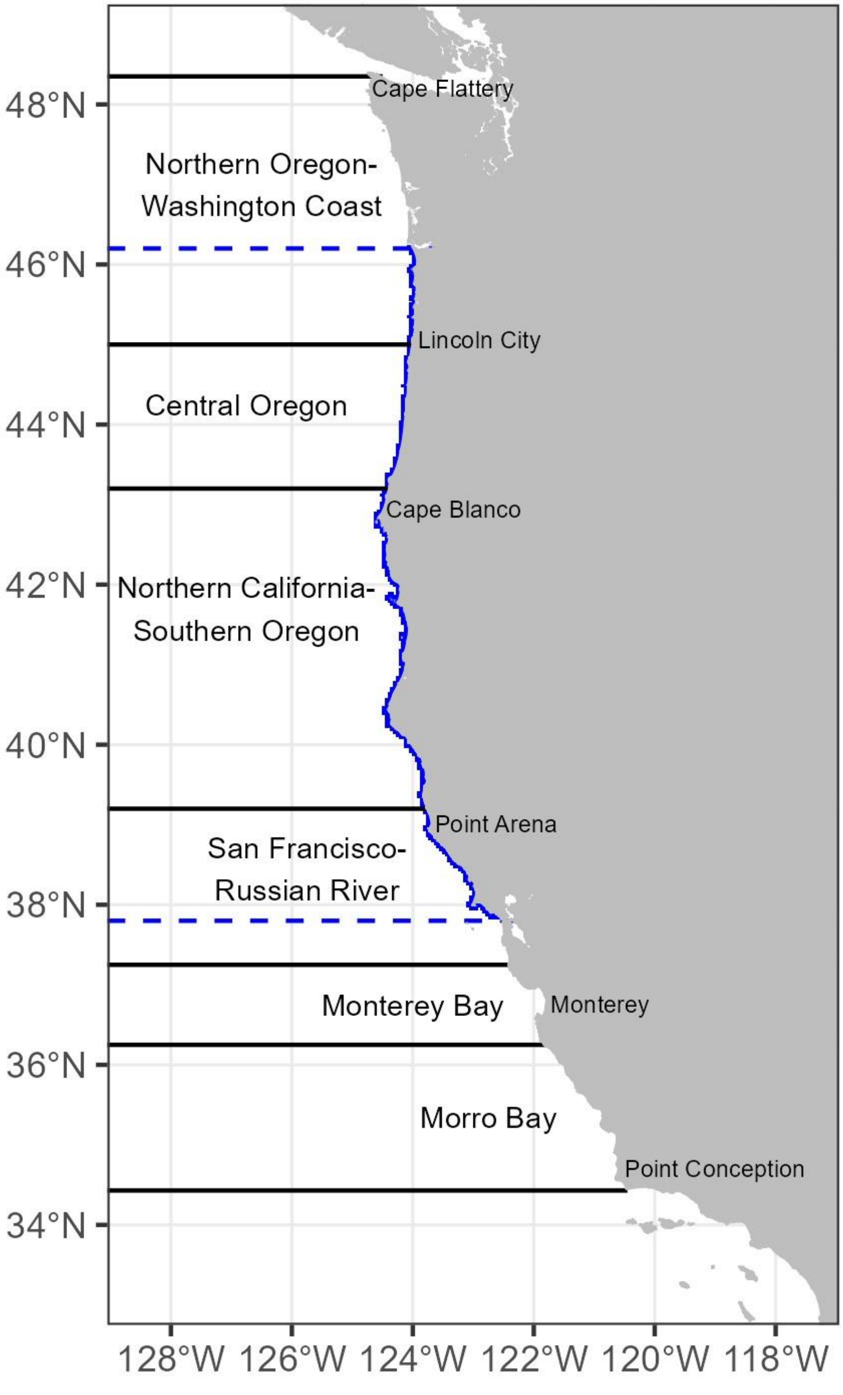

Figure S1. Harbor porpoise stocks along the United States West Coast, with latitudinal boundaries designated by the black horizontal lines. The survey area for this study is shown in the blue polygon, with study area latitudinal boundaries denoted by the dashed blue lines.

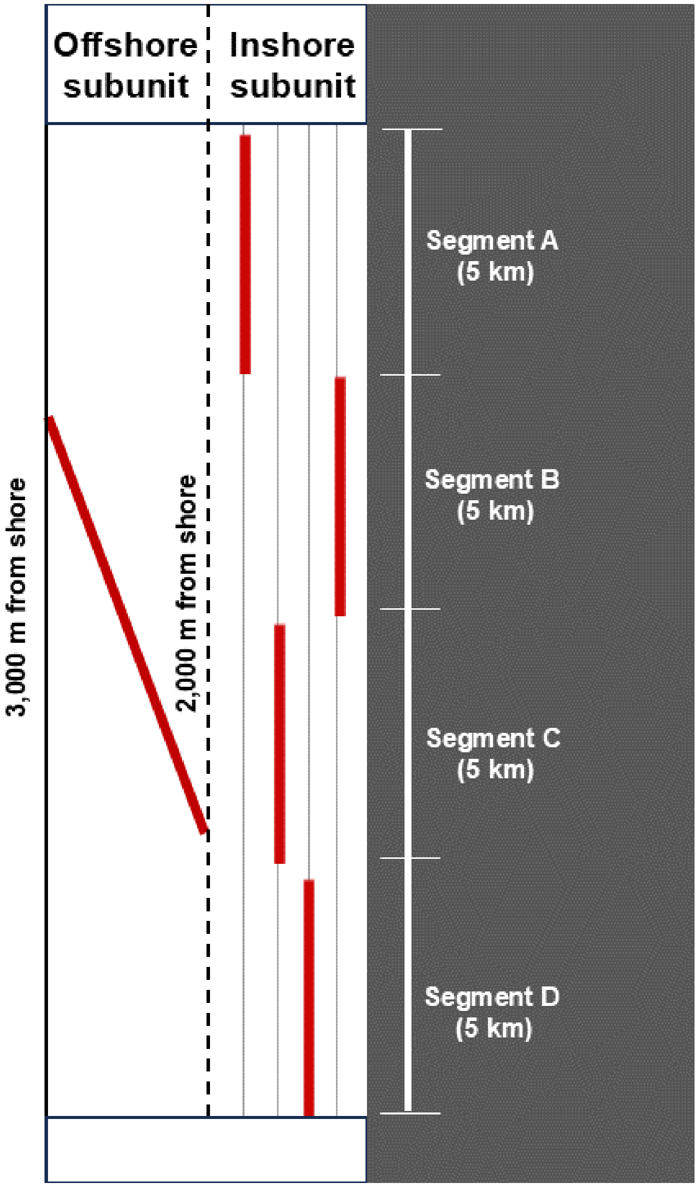

Figure S2. Schematic of the sampling scheme within a primary sampling unit (PSU). The shoreline is denoted by the dark gray. Survey segments are illustrated as the red lines, in both the inshore and offshore subunits.

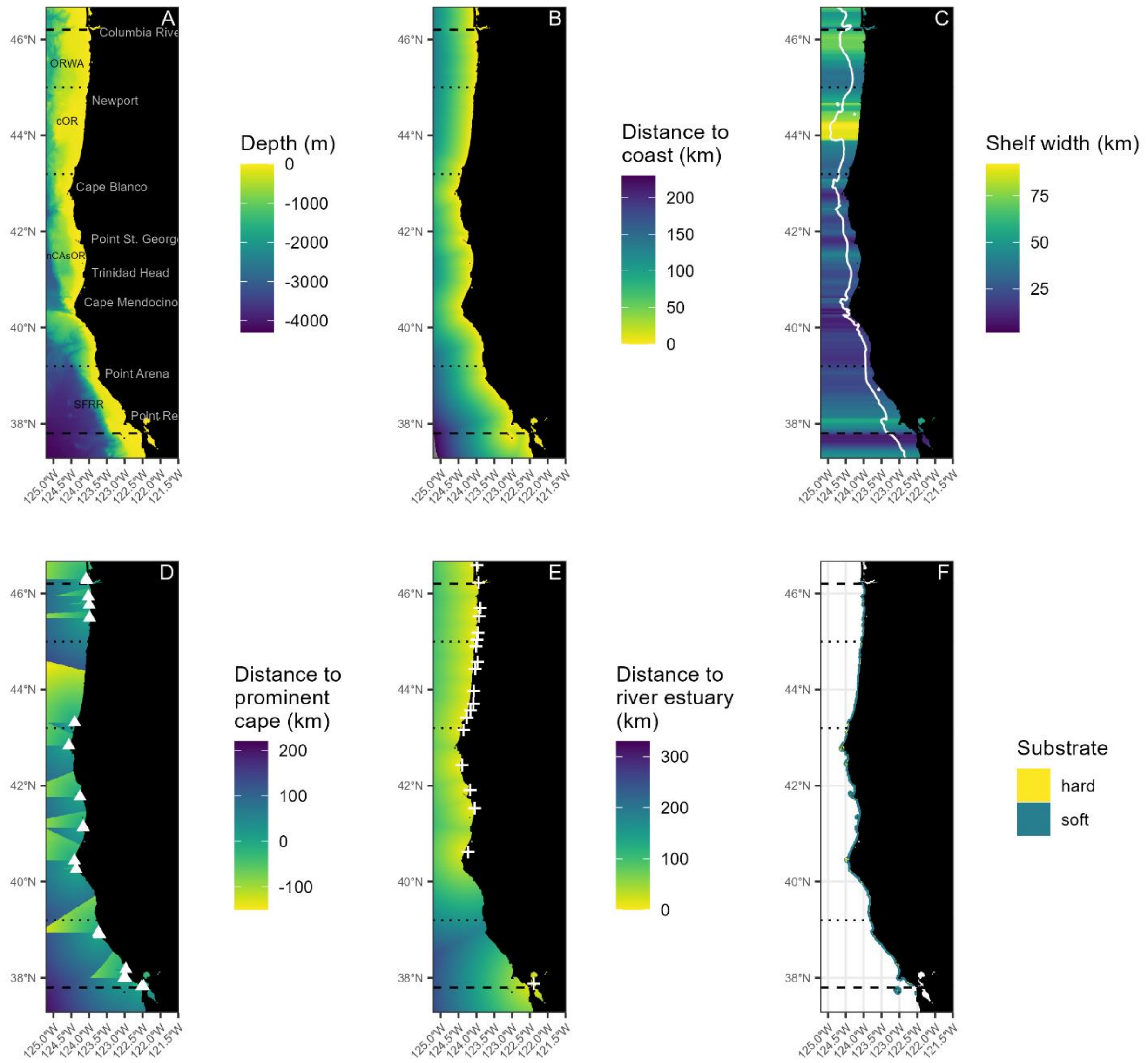

Figure S3. Static environmental predictors included in the harbor porpoise density surface models: A) bathymetric depth, B) distance to the coast, C) distance between the coast and the 200 m isobath, illustrated by the white line, D) distance to the nearest headland, with headlands shown by the white triangles, E) distance to the nearest river estuary, with river estuaries show by the white crosses, and F) benthic substrate type.

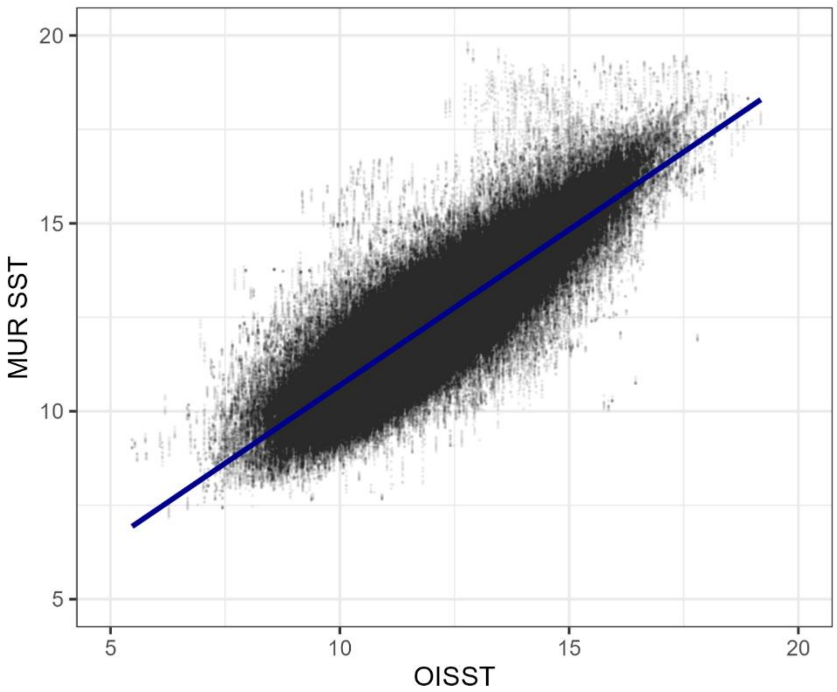

Figure S4. Comparison of daily Optimal Interpolation Sea Surface Temperature (OISST, 0.25° resolution) and Multi-spectral Ultra-high Resolution Sea Surface Temperature (MUR SST, 1 km resolution) sampled at all grid cells within our nearshore study area between 15 May and 15 August of every year from 2002-2022. The two satellite data products are significantly correlated (Pearson's correlation coefficient = 0.86, $p < 2.2*10^{-16}$)

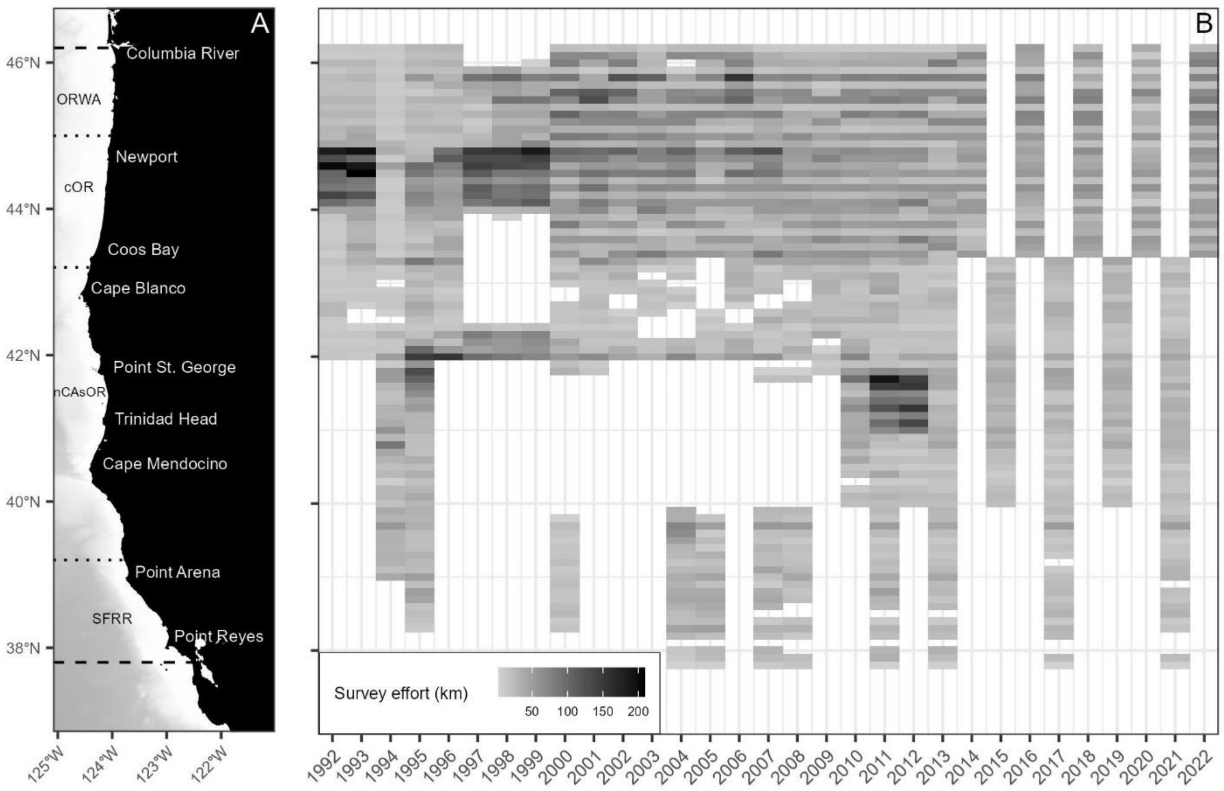

Figure S5. A) Map of the study area, with study area boundaries shown by the dashed lines, harbor porpoise stock boundaries shown by the dotted lines, and major placenames denoted. B) Heatmap illustrating the survey effort throughout the study period, where the y-axis corresponds to the latitude on the map, the x-axis corresponds to the study year, and the fill color indicates the survey effort in km with darker cells indicating higher effort.

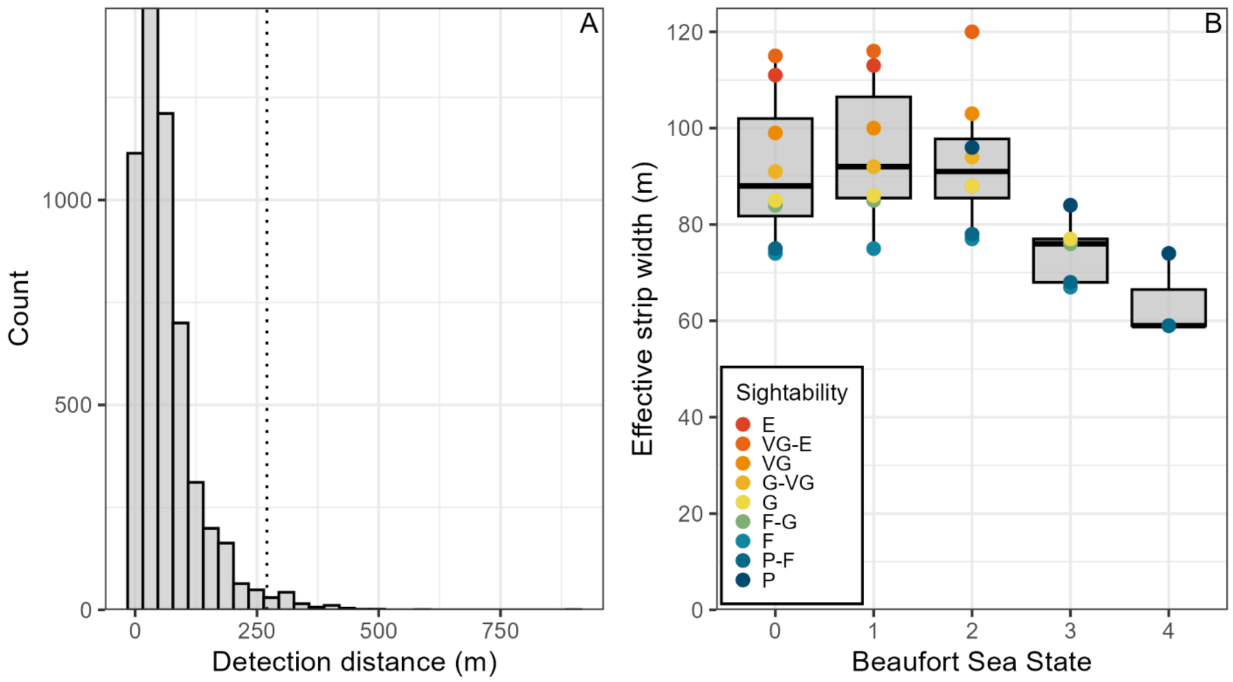

Figure S6. A) Histogram of perpendicular distances from the vessel trackline to harbor porpoise detections. The truncation distance at the 98th percentile is denoted by the black dotted line. B) The effective strip width (ESW) for detecting harbor porpoise under different observation conditions. Points show ESW estimates for each sightability category, and boxes summarize the distribution (median and interquartile range) of ESW across sightability categories for each Beaufort sea state.

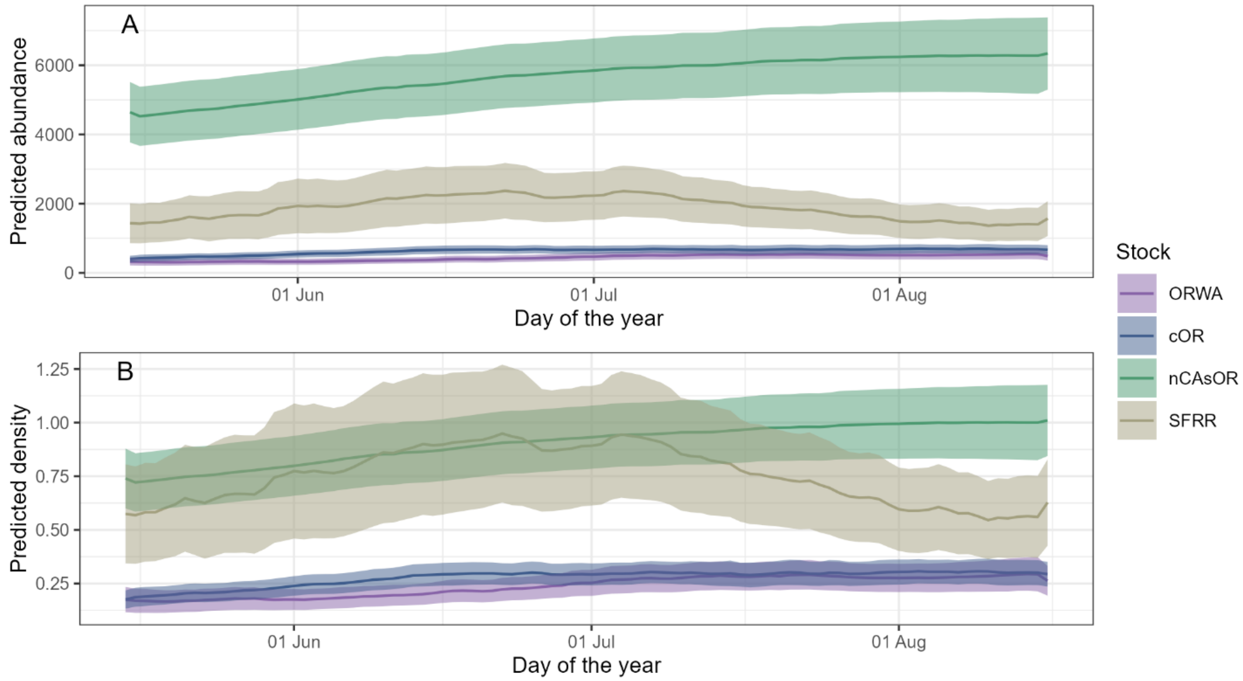

Figure S7. Mean daily predicted abundance (A) and density (B) for each stock, for each day of the study season (15 May through 15 August) across all 31 years of the study (1992-2022). Shading represents the standard error, computed using the coefficient of variation resulting from the uncertainty in the density surface model predictions.

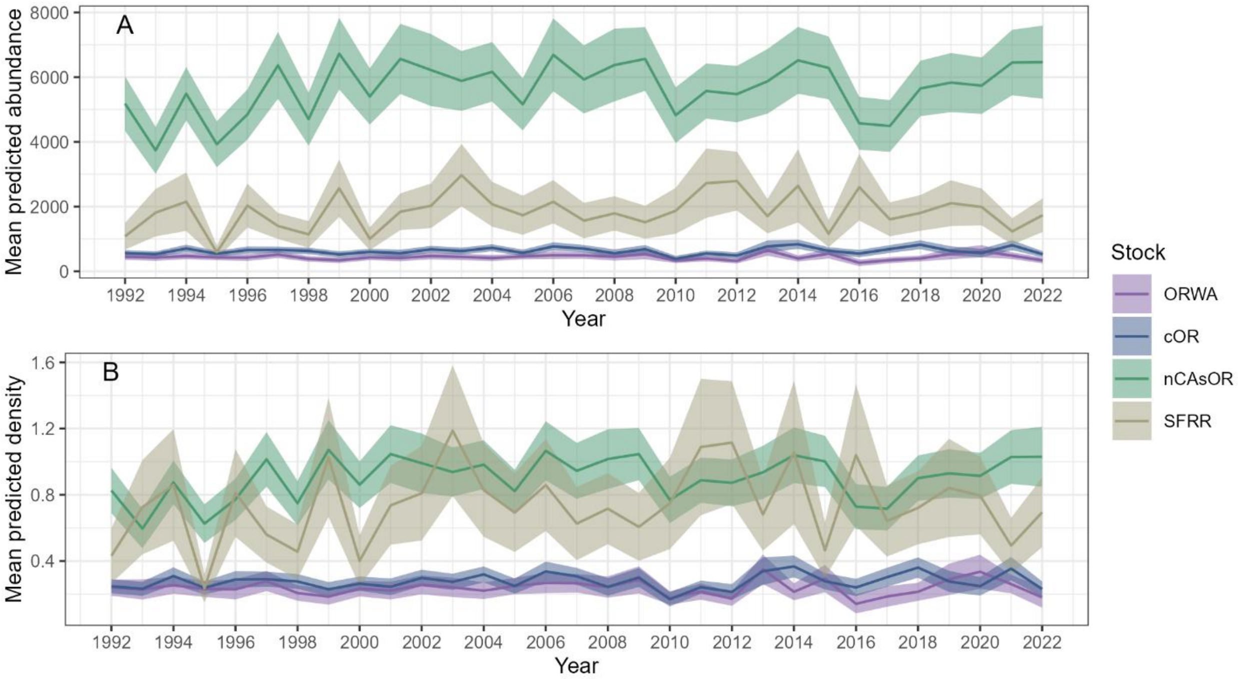

Figure S8. Mean annual predicted abundance (A) and density (B) for each stock across all 31 years of the study (1992-2022). Shading represents the standard error, computed using the coefficient of variation resulting from the uncertainty in the density surface model predictions.

Table S1. Comparison of candidate detection function models, fitted with different covariates (none, Beaufort sea state, overall sightability) and different key functions (hazard-rate, half-normal). Models were compared using the difference in Akaike's information criterion (ΔAIC, lower value indicates better relative performance), and the realism of the predicted effective strip width (ESW) under different covariate conditions.

Covariates	Key function	ΔAIC	ESW range	Median ESW
BSS + Sightability	Hazard-rate	0	59 – 120	99
BSS	Hazard-rate	70.84	66 – 104	98
Sightability	Hazard-rate	179.14	69 – 115	100
BSS + Sightability	Half-normal	222.03	40 – 115	104
Null	Hazard-rate	292.71	95	95
BSS	Half-normal	300.47	53 – 108	103
Sightability	Half-normal	403.80	54 – 114	104
Null	Half-normal	555.22	100	100
